# Nationwide epidemiologic study for fibrosing interstitial lung disease (F-ILD) in South Korea: a population-based study

**DOI:** 10.1186/s12890-023-02373-z

**Published:** 2023-03-22

**Authors:** Kyung-In Joung, Hyemin Park, Sunyoung Park, Ju-Young Shin, Yong Hyun Kim

**Affiliations:** 1 School of AI Healthcare, CHA University, Pocheon, Republic of Korea; 2 VIAplus, Suwon, Republic of Korea; 3 School of Pharmacy, Sungkyunkwan University, 2066 Seobu-ro, Jangan-gu, Suwon, Gyeonggi-do 16419 Republic of Korea; 4 Division of Pulmonary and Critical Care Medicine, Department of Internal Medicine, Bucheon St. Mary’s Hospital, College of Medicine, The Catholic University of Korea, Seoul, Republic of Korea

**Keywords:** Fibrosing interstitial lung disease (F-ILD), Progressive fibrosing-interstitial lung disease (PF-ILD), Idiopathic pulmonary fibrosis (IPF), Prevalence, Incidence, Fatality

## Abstract

**Background:**

Fibrosing interstitial lung disease (F-ILD) is a major public health concern due to its poor prognosis. Recent clinical evidence shows that antifibrotic approaches such as pirfenidone and nintedanib provide better clinical outcome prediction in idiopathic pulmonary fibrosis (IPF) as well as selected progressive fibrosing ILD (PF-ILD) patients. Having epidemiologic insight into these diseases will be essential for the efficient utilization of these therapeutic resources. This study aimed to estimate the current prevalence, incidence, and mortality of F-ILD classified as idiopathic pulmonary fibrosis (IPF), PF-ILD other than IPF, and non-progressive F-ILD and their temporal trend in Korea.

**Methods:**

Population-based retrospective cohort study was conducted using the Korean Health Insurance Review and Assessment (HIRA) database (2011–2018). Patients with IPF were identified using ICD-10 code, RID code, and differential diagnosis approach. By leveraging medical records available from claim data and referencing those used in clinical trials, rigorous diagnostic criteria for PF-ILD detection were implemented.

**Results:**

For the past eight years, the prevalence of IPF and PF-ILD has progressively increased, while non-progressive F-ILD has remained stable. IPF, PF-ILD, and non-progressive F-ILD prevalence per 100,000 in 2018 were 16.9, 10.4, and 11.7, respectively. The incidence of IPF in 2018 was more than twice that of 2012. The incidence of PF-ILD in 2018 was 1.5 times higher than that in 2012. In 2018, the mortalites were 10.3% and 12.2% for IPF and PF-ILD, respectively. The mortality rate of PF-ILD was greater than that of IPF in all years. Unclassifiable PF-ILD and rheumatoid arthritis-PF-ILD had the highest proportion and mortality among the PF-ILD subtypes.

**Conclusion:**

The prevalence and incidence of IPF and PF-ILD have been steadily increasing in recent years. The mortality rate of PF-ILD remained consistently high and exceeded those of IPF in all years.

**Supplementary Information:**

The online version contains supplementary material available at 10.1186/s12890-023-02373-z.

## Background

Interstitial lung diseases (ILDs) refer to a heterogeneous group of parenchymal lung diseases that involve a number of common clinical and pathophysiological features while having a wide range of etiologies and prognoses. The most common type of ILD is idiopathic pulmonary fibrosis (IPF) and is considered as the prototype progressive-fibrosing ILD characterized by a decline in pulmonary function and poor prognosis with a median survival of 3–5 years [1, 2].

In addition to IPF, a number of fibrosing ILDs can exhibit a progressive phenotype during the course of disease resulting in a decline in lung function and early mortality. For this group of fibrosing ILD with a progressive phenotype, the term progressive fibrosing ILD (PF-ILD) have been used widely in clinical field. But there is no standardized definition of progression and the criteria to define PF-ILD are variable in clinicians and researchers. Recently, instead of PF-ILD, the term progressive pulmonary fibrosis (PPF) is suggested and a consensus definition of PPF is determined in the 2022 ATS/ERS/JRS/ALAT clinical guideline. It is defined as at least two of the following three criteria occurring within the past year (1. worsening respiratory symptoms; 2. physiological evidence of disease progression, as defined decline of lung function; and 3. radiological evidence of disease progression) [3].

Due to morphological overlap and shared pathological traits, IPF and PF-ILD, which are subtyped according to their etiology, fall under the concept of progressive fibrosis phenotype. However, there are pathological distinctions between the two. IPF is primarily a fibrotic ILD, whereas in PF-ILD, fibrosis frequently occurs before or is associated with inflammation and the inflammation pathway results in an extracellular matrix that transforms healthy lung tissue into pulmonary fibrosis [4, 5].

Although the disease burden by a poor prognosis of IPF and PF-ILD other than IPF (hereafter referred to as PF-ILD) is a major public health concern, epidemiological studies on these disorders are scarce and need to be updated [6–8]. In case of IPF, although epidemiologic features are well documented, most of them were conducted before 2011, when significant revise of diagnostic criteria was published [6, 9]. The epidemiologic reality of PF-ILD has yet to be fully elucidated, owing to the heterogeneity and uncertainty of diagnosis, and few real-world data based on the population is available [10]. Two recent retrospective cohort studies on incident PF-ILD were for hospital referrals [11, 12]. Recently a French study described epidemiology of patients with PF-ILD using an algorithm for extracting claim data [13]. While another study using claim data employed the frequency of pulmonologist visits as a criterion for defining progression [14]. It has been raised that the narrow definition of IPF and the granular PF-ILD subgrouping may negatively affect the treatment outcome, which further emphasizes the need to closely examine the prognosis by these subgroups [15].

Recent clinical evidence shows that antifibrotic approaches such as pirfenidone and nintedanib guarantee better clinical outcome prediction in IPF as well as selected PF-ILD patients [16–19]. Subsequently, nintedanib has been approved for PF-ILD in the US, European Medicine Agency (EMA), and Korea, [20–22] anticipating to fulfill an unmet treatment need in patients suffering from these life-threatening lung diseases [20]. Therefore, having epidemiologic insight on these diseases will be essential for the efficient utilization of these therapeutic resources. Some previous studies confirmed that the size of IPF and PF-ILD is increasing, but most of the data are before 2011, and the case definition in many studies is not based on the current guideline [9]. There is a need to update the evidence regarding recent epidemiologic patterns in these diseases. Further, regarding that IPF is associated with a genetic predisposition and epigenetic effect, [23] insufficient evidence in Asia underscore the need for this study.

As far as we know, there has been no study that investigates the comprehensive epidemiology and temporal variation of overall F-ILD by well-defined subtype for the entire national population. The purpose of this study was to estimate the prevalence, incidence, and mortality of comprehensive F-ILD classified as IPF, PF-ILD, and non-progressive F-ILD, progression rate among F-ILD other than IPF, and their temporal trend nationwide.

## Methods

### Data source

The Korean Health Insurance Review and Assessment (HIRA) database was used in this study. South Korea has a mandatory single universal health coverage system (National Health Insurance System, NHIS) that the NHIS covers around 98% of the whole Korean population since 2000. HIRA is a government-affiliated organization that reviews and evaluates healthcare costs and healthcare service quality. The HIRA hold the comprehensive database containing all health care utilization information. (https://www.hira.or.kr/rd/insuadtcrtr/InsuAdtCrtrList.do?pgmid=HIRAA030069000400). HIRA database included basically the whole Korean population (97%), unless participants’ eligibility was disqualified due to death or emigration. It provides information on details of all medical utilization including demographic characteristics, inpatients and out-patients services, diagnosis using international Classification of Disease, 10th revision (ICD-10) procedures, and prescription drugs. Individuals are “de-identified” as the database did not contain personal identifying information.

### Study design and population

This was a retrospective cohort study to estimate the prevalence, incidence, and mortality of F-ILD. The eligible study population was all enrollees in the HIRA national database for the period from January 1, 2011 to December 31, 2018.

### Selection of cases

Patients with F-ILD were defined as those aged ≥ 18 years on the index date and had at least one lung disease diagnosis and one F-ILD diagnosis (ICD-10: J84.1), or at least two F-ILD diagnosis (ICD-10: J84.1) for the year based on ICD-10 diagnostic codes. For each year, the index date was defined as the first date that satisfies the case definition by the disease spectrum. Although there was no F-ILD diagnosis in the corresponding year, patients with F-ILD diagnosis in the previous one or two years and who have medical utilization in the year were also included in the case. Next, patients with the IPF, considered as a prototype of ILD, were first selected for those having IPF diagnosis (ICD-10: J84.18) and rare intractable disease (RID) code (V236) simultaneously, and having no differential diagnosis. NHIS has been operating a RIDs registration program since 2006, and IPF belongs to RIDs from 2011. Patients registered as RIDs receive a copayment reduction of up to 90%. Thus, diagnosis and billing for them are stricter in terms of insurance finances, making the data more reliable [24–26].

**Fig. 1:**
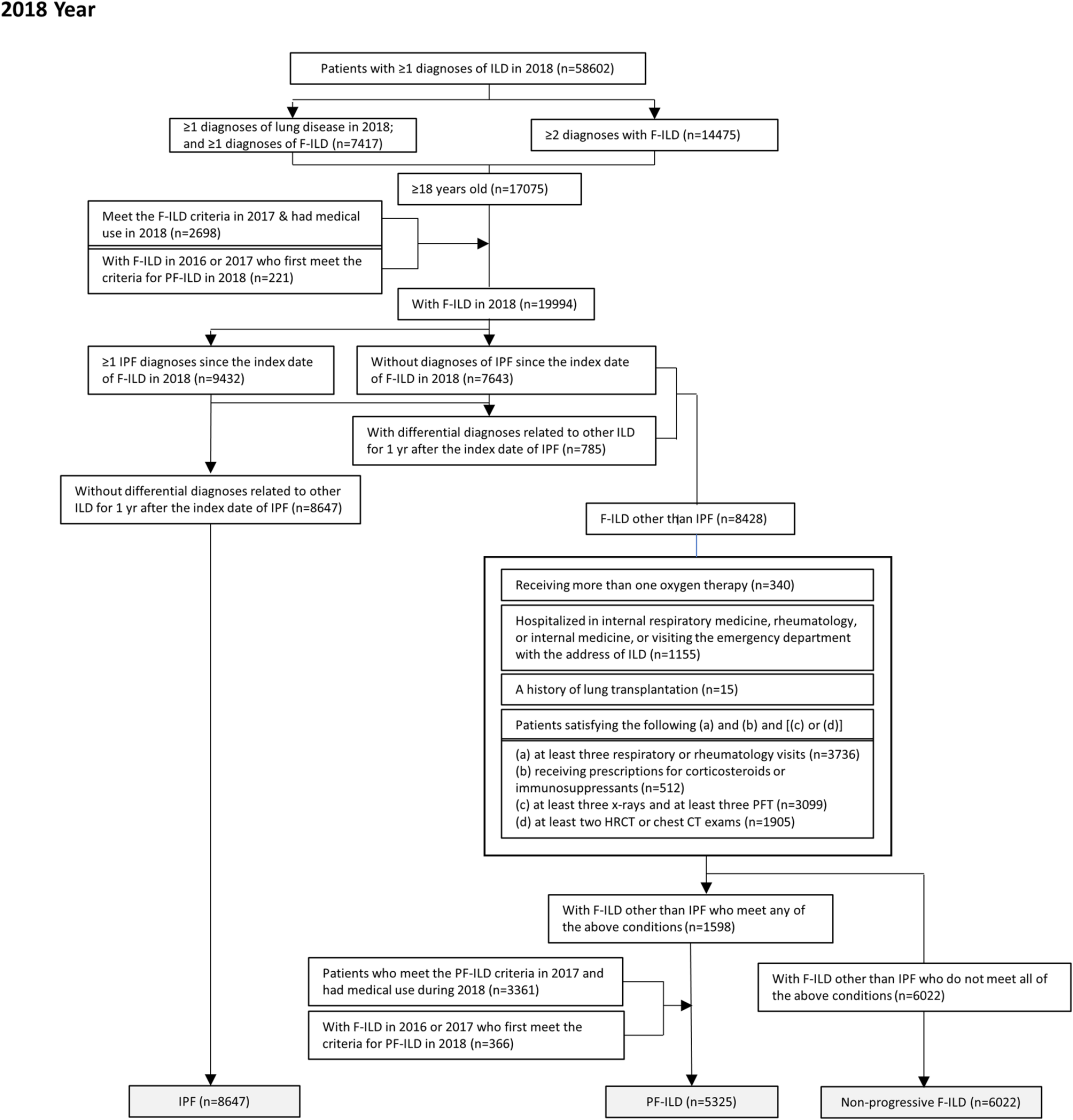
Flow chart for patient selection algorithm in 2018

For selecting PF-ILD patients, an alternative algorithm verifying the progression was developed by referencing INBUILD study and PROGRESS study [11, 13, 19, 27]. Among non-IPF ILD people, patients who met one of the following four criteria for PF-ILD [(1)~(4)] was classified as PF-ILD: (1) receiving more than one oxygen therapy, or (2) being hospitalized in internal respiratory medicine, rheumatology, or internal medicine, or visiting the emergency department with the address of ILD, or (3) a history of lung transplantation, or (4) satisfying the following (a) and (b) and [(c) or (d)] for medical record base on the claim data were included in the PF-ILD: (a) at least three respiratory or rheumatology visits, (b) receiving prescriptions for corticosteroids or immunosuppressants, (c) at least three x-rays and at least three pulmonary function test (PFT), (d) at least two high-resolution computed tomography (HRCT) or chest computed tomography (CT) exams. All of the above medical utilizations were examined from the individual F-ILD index date (the date first identified as F-ILD) to December 31 of the corresponding year. Patients who met the PF-ILD criteria in the previous year and had medical use during the year were also defined as PF-ILD patients in that year. All patients screened for F-ILD were followed up for up to 2 years for PF-ILD inclusion. Respiratory specialists were consulted on the overall setting of patient selection criteria. The remaining patients who did not meet these criteria were assigned to the non-progressive F-ILD group. Figure 1 shows the detailed patient selection algorithm using 2018 as an example.

Index date, the first date that satisfies the case definition by the disease spectrum; F-ILD, fibrosing-interstitial lung disease; IPF, idiopathic pulmonary fibrosis; PF-ILD, progressive fibrosing- interstitial lung disease; PFT, pulmonary function test; HRCT, high-resolution computed tomography; CT, computed tomography.

The subtypes of both PF-ILD and non-progressive F-ILD were divided into hypersensitivity pneumonitis-ILD, autoimmune diseases-ILD (rheumatoid arthritis-ILD, systemic sclerosis-ILD, other connective tissue disease (CTD)-ILD including Sjogren syndrome, and Systemic Lupus Erythematosus (SLE) or dermatomyositis-ILD), sarcoidosis-ILD, ILD due to external factors, and patients whose subtype of ILD could not be identified as unclassifiable ILD. These subtypes were identified using corresponding diagnostic code during 180 days prior to the index date (supplementary Table 1).

### Measurement and analysis

Descriptive statistics were used to summarize the characteristics of patients with IPF, PF-ILD, and non-progressive F-ILD including sex, age, type of insurance, type of institution for each year based on the specifications of the index date. Charlson Comorbidity Index (CCI) was measured using the diagnosis history for 12 months prior to the index date. The chi-square test or Fisher’s exact test was used to assess the difference between the disease subgroups.

Patients who met each disease group’s criteria stated in the ‘Selection of cases’ section above were considered prevalent cases. Prevalence was calculated by dividing the number of cases by the total population in South Korea. To be included as an incident case, the patient in the current year must not have been selected in the previous calendar year. Incidence rate was calculated by dividing the number of incident cases by the population at risk. Case mortality was determined by dividing the number of deaths by the number of prevalent cases. The mid-year population was used for the whole domestic population, with data from the Korea Statistical Information Service (KOSIS) [28]. Prevalence and incidence were expressed as the number of cases/100000 persons. The risk of progression among F-ILD other than IPF was calculated by dividing the number of PF-ILD patients by the number of F-ILD other than IPF patients.

### Sensitivity analysis

As part of the sensitivity test, in order to assign PF-ILD patients to subgroups, instead of checking for ICD-10 codes corresponding to subtypes until 180 days before the index date, ICD-10 code in the year of the first diagnosis of PF-ILD was used as a criterion for subtype determination.

All data were analyzed using the SAS statistical application program (Version 9.4, SAS Institute Inc, NC, USA). The study protocol was approved by Sungkyunkwan University’s Institutional Review Board (IRB No. 2020-03-001). Informed consent was waived by the Sungkyunkwan University’s Institutional Review Board.

## Results

Table 1 shows the general characteristics of patients with F-ILD in 2018. The total number of F-ILD patients was 19,994, with IPF, PF-ILD, and non-progressive F-ILD comprising 43.3%, 26.6%, and 30.1%, respectively. While not all are provided, the basic demographics by disease type in 2011 to 2017 were similar to those in 2018, and were comparable with previous studies [29, 30]. In all disease types the average age of patients was about 70 years old, and the proportion of elderly patients aged 70 years or older was more than 50%. IPF was found to be approximately three times more prevalent in men than in women while PF-ILD was observed to be equally prevalent in both sexes.

**Table 1:** General characteristics of interstitial lung disease in 2018

Characteristic	Overall Fibrosing-Interstitial lung disease (F-ILD)	Disease Type			*p-value*
		Idiopathic pulmonary fibrosis (IPF)	Progressive fibrosing-interstitial lung disease (PF-ILD)	Non progressive fibrosing-interstitial lung disease (non-progressive F-ILD)	
	19,994 (100%)	8647 (43.3%)	5325 (26.6%)	6022 (30.1%)	
Age					
Mean (SD)	70.6 (10.7)	71.92 (8.7)	69.2 (12.0)	69.8 (11.8)	< 0.0001
18–29	49 (0.2)	4 (0.0)	19 (0.4)	26 (0.4)	
30–39	192 (1.0)	14 (0.2)	89 (1.7)	90 (1.5)	
40–49	544 (2.7)	76 (0.9)	248 (4.7)	220 (3.7)	
50–59	2060 (10.3)	597 (6.9)	712 (13.4)	750 (12.5)	
60–69	5432 (27.2)	2514 (29.1)	1386 (26.0)	1539 (25.6)	
70–79	7754 (38.8)	3767 (43.6)	1814 (34.1)	2171 (36.1)	
≥ 80	3963 (19.8)	1675 (19.4)	1057 (19.8)	1226 (20.4)	
Sex					< 0.0001
Male	12,830 (64.2)	6434 (74.4)	2784 (52.3)	3612 (60.0)	
Female	7164 (35.8)	2213 (25.6)	2541 (47.7)	2410 (40.0)	
Type of health insurance					< 0.0001
Health Insurance	18,021 (90.1)	8177 (94.6)	4626 (86.9)	5246 (87.1)	
Medical Aid	1973 (9.9)	470 (5.4)	699 (13.1)	776 (12.9)	
Type of institution					< 0.0001
Tertiary general hospital	9862 (49.3)	5554 (64.2)	2457 (46.1)	1958 (32.5)	
General hospital	6885 (34.4)	2810 (32.5)	1637 (30.7)	2414 (40.1)	
Hospital	1459 (7.3)	101 (1.2)	458 (8.6)	852 (14.1)	
Clinic	1788 (8.9)	182 (2.1)	773 (14.5)	798 (13.3)	
CCI					
Mean (SD)	3.73 (2.61)	3.44 (2.3)	3.99 (2.8)	3.81 (2.7)	
CCI					< 0.0001
0	837 (4.2)	359 (4.2)	239 (4.5)	244 (4.1)	
1	2904 (14.5)	1420 (16.4)	638 (12.0)	867 (14.4)	
≥ 2	16,253 (81.3)	6868 (79.4)	4448 (83.5)	4911 (81.6)	

The prevalence by year from 2011 to 2018 were summarized in Table 2. In 2018, the overall prevalence of F-ILD, IPF, PF-ILD, and non-progressive F-ILD were 39.0, 16.9, 10.4, and 11.7 per 100,000 people, respectively. The prevalence of IPF and PF-ILD gradually increased with the year. Compared with 2011, the prevalence of IPF and PF-ILD in 2018 was 2.8 times and 4.3 times higher, respectively. In contrast, the prevalence of non-progressive F-ILD remained almost the same for eight years.

**Table 2:** Prevalence of fibrosing interstitial lung disease by year according to disease classification, 2011–2018

		Overall fibrosing interstitial lung disease (F-ILD)		Idiopathic progressive fibrosis (IPF)		Progressive fibrosing-interstitial lung disease (PF-ILD)		Non-progressive fibrosing-interstitial lung disease	
Year	Number of population	Number of cases	Prevalence per 100,000	Number of cases	Prevalence per 100,000	Number of cases	Prevalence per 100,000	Number of cases	Prevalence per 100,000
2011	50,111,476	9433	18.82	3065	6.12	1216	2.43	5152	10.28
2012	50,345,325	11,295	22.44	3553	7.06	1849	3.67	5893	11.71
2013	50,558,952	12,368	24.46	4159	8.23	2273	4.50	5936	11.74
2014	50,763,158	13,422	26.44	4901	9.65	2851	5.62	5670	11.17
2015	50,951,719	14,659	28.77	5653	11.09	3588	7.04	5418	10.63
2016	51,112,972	16,380	32.05	6714	13.14	4265	8.34	5401	10.57
2017	51,230,704	17,679	34.51	7330	14.31	4696	9.17	5653	11.03
2018	51,300,880	19,994	38.97	8647	16.86	5325	10.38	6022	11.74

Table 3 represents the annual incidence rate from 2012 to 2018. The incidences of IPF, PF-ILD, and non-progressive F-ILD per 100,000 people were 6.2, 3.1, and 9.0, respectively. IPF and PF-ILD incidence rates steadily raised, and the incidence rate of IPF and PF-ILD in 2018 was 1.3 times and 2.1 times higher than that in 2012. Non-progressive F-ILD, on the other hand, has remained unchanged for the past eight years.

**Table 3:** Incidence of fibrosing interstitial lung disease by year according to disease classification, 2012–2018

	Overall fibrosing interstitial lung disease (F-ILD)		Idiopathic pulmonary fibrosis (IPF)		Progressive fibrosing-interstitial lung disease (PF-ILD)		Non-progressive- fibrosing-interstitial lung disease (nonPF-ILD)	
Year	Population at risk	Incidence per 100,000	Population at risk	Incidence per 100,000	Population at risk	Incidence per 100,000	Population at risk	Incidence per 100,000
2012	50,336,733	11.84	50,342,572	2.99	50,344,343	1.98	50,340,468	8.90
2013	50,548,601	11.89	50,555,789	3.51	50,557,367	1.79	50,553,349	8.86
2014	50,751,878	11.93	50,759,452	3.94	50,761,200	2.21	50,757,542	8.07
2015	50,939,503	12.36	50,947,323	4.26	50,949,236	2.69	50,946,382	7.66
2016	51,099,586	13.54	51,107,907	5.43	51,109,797	2.81	51,107,826	7.91
2017	51,215,818	13.59	51,224,684	5.19	51,227,002	2.56	51,225,540	8.48
2018	51,284,805	15.72	51,294,297	6.20	51,296,799	3.07	51,295,469	8.99

Table 4 shows the mortality by year. In 2018, the mortality rates for IPF, PF-ILD, and non-progressive F-ILD patients were 10.3%, 12.2%, and 3.4%, respectively. In all years, the mortality of PF-ILD was higher than that of IPF. Looking at the yearly trend, there was little change in both IPF and PF-ILD.

**Table 4:** Mortality of fibrosing interstitial lung disease by year according to disease classification, 2012–2018

Year	Overall fibrosing interstitial lung disease (F-ILD)			Idiopathic pulmonary fibrosis (IPF)			Progressive fibrosing-interstitial lung disease (PF-ILD)			Non-progressive fibrosing-interstitial lung disease		
	Number of cases	Number of death	Mortality rate (%)	Number of cases	Number of death	Mortality rate (%)	Number of cases	Number of death	Mortality rate (%)	Number of cases	Number of death	Mortality rate (%)
2012	11,295	944	8.36	3553	390	10.98	1849	264	14.28	5893	290	4.92
2013	12,368	1088	8.80	4159	453	10.89	2273	315	13.86	5936	320	5.39
2014	13,422	1206	8.99	4901	505	10.30	2851	368	12.91	5670	333	5.87
2015	14,659	1273	8.68	5653	588	10.40	3588	413	11.51	5418	272	5.02
2016	16,380	1494	9.12	6714	694	10.34	4265	563	13.20	5401	237	4.39
2017	17,679	1604	9.07	7330	747	10.19	4696	615	13.10	5653	242	4.28
2018	19,994	1747	8.74	8647	893	10.33	5325	651	12.23	6022	203	3.37

In 2018, among the subtype of PF-ILD identified, the number of patients whose specific disease was unknown accounted for 50% of the cases. Rheumatoid arthritis-ILD was accounted for the next highest proportion (31.4%), followed by other CTD-ILD including Sjogren syndrome (7.9%) and systematic sclerosis-ILD (5.6%). Among non-progressive F-ILDs, 53.7% belonged to unclassified, and most of the classifiable subtypes belonged to rheumatoid arthritis-ILD (39.5%) (Table 5).

**Table 5:** Number and proportion of patients by subtype in F-ILD other than IPF, 2018

Subtype	Progressive fibrosing-interstitial lung disease (PF-ILD)	Non-progressive- fibrosing-interstitial lung disease (nonPF-ILD)
	n = 5325 (%)	N = 6022 (%)
Hypersensitivity pneumonitis-ILD	57 (1.07)	10 (0.17)
Autoimmune diseases-ILD	2484 (46.65)	2682 (44.54)
Rheumatoid arthritis-ILD	1672 (31.40)	2377 (39.47)
Systemic sclerosis-ILD	296 (5.56)	88 (1.46)
Other CTD-ILD including Sjogren syndrome	420 (7.89)	199 (3.30)
Systemic Lupus Erythematosus (SLE) or dermatomyositis-ILD	245 (4.60)	69 (1.15)
Sarcoidosis-ILD	28 (0.53)	13 (0.22)
ILD due to external factors	119 (2.23)	103 (1.71)
Unclassifiable ILD	2669 (50.12)	3231 (53.65)

Abbreviations: F-ILD, fibrosing-interstitial lung disease; IPF, idiopathic pulmonary fibrosis; CTD, connective tissue disease

From 2011 to 2018, the prevalence of PF-ILD increased in all subtypes. In 2018, the prevalence of autoimmune disease-ILD, rheumatoid arthritis-PF-ILD and hypersensitivity pneumonia-PF-ILD were all five times higher than that in 2011. There was no significant difference in non-progressive F-ILD by year. Although data were not provided, there was little difference in the proportion of each subtype by year (Supplementary Table 2).

The progression rate of F-ILD other than IPF calculated by dividing the number of PF-ILD patients by the number of F-ILD other than IPF patients steadily increased from 19.1% to 2011 to 46.9% in 2018. Except for sarcoidosis, all subtypes showed a 2-5-fold increase (Supplementary Table 3).

Among the subtypes of PF-ILD, the incidence rate of rheumatoid arthritis-ILD increased steadily from 2012 to 2018, and the incidence rate per 100,000 people in 2012 was 0.64 cases and 1.19 cases in 2018. On the other hand, the incidence rate of other classified subtypes did not show much difference by year. The incidence per 100,000 in the unclassifiable subgroup increased 1.4 times from 1.07 to 2012 to 1.54 in 2018. Non-progressive F-ILD showed a decreasing trend in systemic sclerosis-ILD, from 0.14 to 2012 to 0.076 in 2018. Incidence rates in other subtypes did not show an annual trend (Supplementary Table 4).

The annual trend of the mortality rate of PF-ILD was different for each subtype. Prevalence of autoimmune diseases-ILD decreased slightly from 13.9% to 2012 to 10.5% in 2018. On the other hand, unclassifiable ILD, which accounts for the largest proportion among subtypes, was remained nearly unchanged from 14.8% to 2012 to 13.8% in 2018, and the mortality rate of sarcoidosis-ILD and ILD due to external factors increased significantly. (Supplementary Table 5).

As part of the sensitivity test, in order to assign PF-ILD patients to subgroups, instead of checking for ICD-10 codes corresponding to subtypes until 180 days before the index date, ICD-10 code in the year of the first diagnosis of PF-ILD was used as a criterion for subtype determination. The results of the sensitivity test using the year of the first diagnosis of PF-ILD as the criterion for subtype determination were not significantly different from the primary results (Supplementary Table 6).

## Discussion

In this cohort study, the epidemiology of F-ILD from 2011 to 2018 was explored in Korea using national data. The prevalence of IPF and PF-ILD increased steadily for eight years, and non-progressive F-ILD was maintained. In 2018, the prevalence of IPF, PF-ILD, and non-progressive F-ILD per 100,000 were 16.9, 10.4, and 11.7, respectively. The incidence of IPF in 2018 was 6.2 per 100,000, more than twice as high as in 2012, and that of PF-ILD in 2018 was 3.1 per 100,000, which was 1.5 times higher than that in 2012. In 2018, the mortality rates were 10.3% and 12.2% for IPF and PF-ILD, respectively. The mortality of PF-ILD was higher than that in IPF in all years.

### Epidemiology of IPF

The prevalence of IPF Korea seems to be similar to or somewhat lower than that of European and North American countries [6, 31, 32]. In order to be defined as IPF in this study, ICD-10 (J84.18) and RID code (V236) should be coexisted and differential diagnosis should be also satisfied. Specifically, compared with the previous studies using such a narrow definition and administrative data, prevalence of IPF in our study seemed somewhat low. In the previous studies in Canada and Italy, it was estimated as 12.6 per 100,000 (2005–2010) and 20 per 100,000 (2007–2011), respectively [31, 32]. In the case of the incidence, a systematic literature review that synthesized heterogeneous studies in terms of study year (1968–2012), data source, and target subject estimated incidence in Europe and North America to be 3–9 persons/100,000 people [33]. The crude incidence in two UK investigations employing a large database from 2000 to 8.7/100,000 and 7.4/100,000, respectively [34, 35]. Given the data year, the incidence in our study (2.99–6.20/100,000, from 2011 to 2018) appears to be slightly lower.

Epidemiologic studies of IPF in Asian countries are rare. As a domestic study targeting 30 years of age or older, the incidence by narrow definition was 1.84 per 100,000 people using data from 1992 to 2000 [36]. In a more recent study, the prevalence and incidence rates were 35/100,000 people and 8.2/10,000 people in 2013, respectively, higher than in our study, which seems to be related to the broader case definition [37]. In a Japanese study, which used medical benefit data, there were 1.22/100,000 cases, but this study was extrapolated from a sample cases, and the data used was from 2005 [38].

While findings on the temporal trend of IPF epidemiology are mixed, [29, 33, 35, 39–41] the majority of them identified a gradual increase in the incidence, [29, 33, 35, 41] and the present study revealed a steady increase in both the incidence and prevalence as well. The trend in the prognosis of IPF are mixed. According to a study that collected death certification data from multiple countries, the global mortality rate in IPF has been steadily increasing [42]. On the other hand, some studies confirmed improvement in survival and stated that this could partially explain the gradual increase in prevalence. [32, 43]. The incidence and prevalence of IPF both increased in the current study, whereas the mortality rate for IPF remained unchanged at 10%. Most earlier research used data before 2011, however, our data used the most recent data up to 2018, implying that there has been no substantial improvement in prognosis recently.

### Epidemiology of PF-ILD

While a large number of non-IPF ILDs develop into a progression phenotype with poor prognosis, there is little epidemiologic research on them [15, 44]. In the present study, the progression rate of F-ILD other than IPF was 19% in 2011, and it gradually increased to 47% in 2018, which was higher than those in previous studies. Real-world data from a hospital-based cohort was used in two recent retrospective observational studies conducted in France and England. Both of them followed the pre-defined criteria of disease progression in the INBUILD trial [27] with the advantage of high validity of the case definition and found the progressive fibrosing phenotype among F-ILD other than IPF was 27.2% (168/617) and 14.5% (253/1749), respectively [11, 12]. According to an analysis of US claim data, 15% of non-IPF ILD patients had PF-ILD. Since the study focused on healthcare consumption and cost, it did not include data on prevalence [14].

In a systematic review that recently combined the published literature providing the prevalence of ILD and survey data to estimate the proportion of progressive phenotype, the overall prevalence of PF-ILD in Europe and the US were estimated to be 2.2–20.0 per 100,000 and 28.0 per 100,000 people, respectively [7]. In our study, the prevalence of PF-ILD was estimated to be 10.4 per 100,000 as of 2018, which is similar to or slightly lower than that of Europe and the US. However, the authors indicated that the estimate of overall and individual ILDs with a progressive fibrosing phenotype was based on an exploratory approach based on a quantitative physician survey to predict the proportions expected to progress, rather than a population-based. Notably, our study used a distinct methodology, including patients with progressive phenotype who were identified using strict operational parameters.

The prevalence and incidence of PF-ILD increased significantly between 2012 and 2018, by 4.3 and 1.6 times, respectively. Despite the possible influx of earlier patients by diagnostic technology like HRCT, [45] the prognosis showed no improvement. The mortality of PF-ILD was higher than that of IPF in all years, suggesting unmet healthcare needs [46].

While direct comparisons between studies are cautious due to differing target groups and subclassification criteria, the proportion of subtypes in PF-ILD also varied from study to study. For example, whereas the proportion of rheumatoid arthritis-ILD was quite high in our study (31% vs. 4%), that of systemic sclerosis was remarkably high in the PROGRESS study. (26% vs. 6%) (11) In the clinical cohort study in the UK, the most common diagnoses associated with a PF-ILD were hypersensitivity pneumonitis-ILD (33.2%), unclassifiable ILD (17.3%), and connective tissue disease-ILDs including rheumatoid arthritis-ILD (16.6%), while our study indicated as 1%, 50%, and 47%, respectively. Connective tissue disease-associated ILD (0.5–10.2 per 100,000 people) and sarcoidosis (1.9–66.1 per 100,000 people) were reported as the most common subtypes in the systematic literature review, [7] while the numbers were figured as 0.8/100,000 and 0.05/100,000, respectively in our study, as of 2018.

The mortality of PF-ILD also varied by subtype, and the high mortality in unclassifiable ILD, which accounts for nearly half of all PF-ILD, is consistent with the previous study [11]. Such a poor prognosis in unclassifiable PF-ILD can be attributed to the lack of diagnostic location and the associated management uncertainty [15, 47]. This study also revealed that rheumatoid arthritis-PF-ILD was a specific burden in Korea. Besides, the development of ILD in rheumatoid arthritis has been linked to a three-fold increased mortality [48]. Special efforts should be given to improve survival in unclassifiable-ILD and rheumatoid arthritis-ILD.

Additionally, we found that even three years after antifibrotic agents such as pirfenidone and nintedanib were introduced, half of the patients with IPF did not receive antifibrotic treatment. Out of 8647 IPF patients in 2018, only 4220 patients had received a prescription for antifibrotic agents within one year after the index date. It is also reported that over one-third of PF-ILD patients were not receiving any medication [49]. It indicates that there is a major unmet need in the treatment of both IPF and PF-ILD.

### Strengths and limitations

This study has several strengths. First, this is the first epidemiologic study to investigate the prevalence, incidence, and mortality rates of IPF, PF-ILD, and non-progressive F-ILD throughout the entire national population. Due to the rarity of these disorders, this population-based approach is thought to be particularly suitable for better estimation. Second, our study tried to minimize diagnostic misclassification by making the operational definition to find the progressive phenotype in the claim data aligning to that used in the clinical trials [19, 50]. Third, although there are criticisms on the accuracy of the diagnostic code recorded in the claim data, the NHIS in Korea conducts a stringent insurance review for RID registration in the case of IPF, so the case definition for IPF in this study seems to be reliable. In a study on the epidemiology of inflammatory bowel disease, RID codes had a sensitivity and specificity of 97–98% and 93–94%, respectively [24]. Finally, temporal observation of disease occurrence is critical for disease prevention and management. Our study identified the trend by tracking epidemiologic data for 8 years until 2018 and revealed that both the prevalence and the incidence of IPF and PF-ILD continued to increase. Furthermore, when evidence on the influence of genetic and epigenetic variables is being added to F-ILD, it is also important to include data from Asian nations that are now deficient.

The present study has some limitations. First, while the case was carefully defined utilizing all medical records available from the claim data, verification of its accuracy is a future task. Specifically, it cannot be ruled out that the high proportion of unclassifiable F-ILD in this study may have attributed to the subtyping algorithm developed based on medical records available from claim data. Second, this study did not perform a stratification analysis based on basic demographic category such as gender and age. In addition, co-morbidities or medications that could affect the development and prognosis of F-ILD were not considered. Future research incorporating these factors will be needed. Finally, there were changes such as the introduction of the antifibrotic agent in 2015 and evidence supporting antifibrotic therapy in PF-ILD are adding [16, 19] Investigating the mortality by pretreatment will be another topic for future study.

Korea’s claim data includes information on the entire national citizen’s medical use, allowing for long-term follow-up. Survival analysis is available since the data is not segmented. These considerations suggest that the utilization of these data for rare disorders like F-ILD has greater utility because all cases can theoretically be captured. If future claims data are used to evaluate survival according to treatment decision for each illness category of F-ILD, it will be valuable real-world evidence for reducing the healthcare burden in terms of clinical and public health.

## Conclusion

In a population-based study to investigate the epidemiology of F-ILD in Korea for the past 8 years, the disease burden of IPF and PF-ILD was comparable to or slightly lower than that of European and North American countries. The incidence and prevalence of IPF and PF-ILD have gradually increased since 2011. The mortality rate of PF-ILD was consistently high and exceeded those of IPF in all years emphasizing unmet healthcare needs. The epidemiology of PF-ILD varied depending on the subtype showing a remarkably high proportion and mortality in unclassifiable PF-ILD and rheumatoid arthritis-PF-ILD.

## Electronic supplementary material

Below is the link to the electronic supplementary material.

Supplementary Material 1

## Data Availability

The data that support the findings of this study are available from the Health Insurance Review & Assessment Service in Korea, but restrictions apply to the availability of these data, which were used under license for the current study, and so are not publicly available. Data are however available from the authors upon reasonable request and with permission of the Health Insurance Review & Assessment Service in Korea.

## List of abbreviations

F-ILD: fibrosing interstitial lung disease
PF-ILD: progressive fibrosing-ILD
IPF: idiopathic pulmonary fibrosis
HIRA: Korean Health Insurance Review and Assessment
PPF: progressive pulmonary fibrosis
EMA: European Medicine Agency
NHIS: National Health Insurance System
RID: rare intractable disease
HRCT: high-resolution computed tomography
CT: chest computed tomography
PFT: pulmonary function test
CTD: connective tissue disease
CCI: Charlson Comorbidity Index
KOSIS: Korea Statistical Information Service
