# Supplementary material for: Nationwide epidemiologic study for fibrosing interstitial lung disease (F-ILD) in South Korea: a population-based study
Source: BMC Pulm Med. 2023 Mar 22;23:98. doi: 10.1186/s12890-023-02373-z (PMC10035232; doi:10.1186/s12890-023-02373-z)
Supplement: Supplementary file 1 — Supplementary Material 1 [file 12890_2023_2373_MOESM1_ESM.docx]

Supplementary Table 1. International classification of disease code by subtype of fibrosing-interstitial lung disease.

| Subtype | ICD-10 | description |
| --- | --- | --- |
| Hypersensitivity pneumonitis-ILD | J67 | Hypersensitivity pneumonitis due to organic dust |
| Autoimmune diseases-ILD |  |  |
| Rheumatoid arthritis-ILD | M05 | Seropositive rheumatoid arthritis |
|  | M06 | Other rheumatoid arthritis |
|  | M12 | Other specific arthropathies |
|  | M13 | Other arthritis |
| Systemic sclerosis-ILD | M34 | Systemic sclerosis |
| Other CTD-ILD including Sjogren syndrome | M35 | Other systemic involvement of connective tissue |
| Systemic Lupus Erythematosus (SLE) or dermatomyositis-ILD | M32 | Systemic lupus erythematosus |
|  | M33 | Dermatopolymyositis |
| Sarcoidosis-ILD | D86 | Sarcoidosis |
| ILD due to external factors | J60 | Coalworker’s pneumoconiosis |
|  | J61 | Pneumoconiosis due to asbestos and other mineral fibres |
|  | J62 | Pneumoconiosis due to dust containing silica |
|  | J63 | Pneumoconiosis due to other inorganic dusts |
|  | J64 | Unspecified pneumoconiosis |
|  | J66 | Airway disease due to specific organic dust |
|  | J70 | Respiratory conditions due to other external agents |

Abbreviations: ILD, interstitial lung disease; CTD, connective tissue disease

Supplementary Table 2. Prevalence by subtype in F-ILD other than IPF, 2011-2018

| Subtype | Prevalence per 100,000 | | | | | | | |
| --- | --- | --- | --- | --- | --- | --- | --- | --- |
|  | 2011 | 2012 | 2013 | 2014 | 2015 | 2016 | 2017 | 2018 |
| Progressive fibrosing-interstitial lung disease (PF-ILD) | | | | | | | | |
| Hypersensitivity pneumonitis-ILD | 0.02 | 0.03 | 0.05 | 0.07 | 0.12 | 0.12 | 0.11 | 0.11 |
| Autoimmune diseases-ILD | 1.00 | 1.49 | 1.86 | 2.36 | 3.18 | 3.84 | 4.21 | 4.84 |
| Rheumatoid arthritis-ILD | 0.76 | 1.07 | 1.30 | 1.63 | 2.23 | 2.62 | 2.83 | 3.26 |
| Systemic sclerosis-ILD | 0.08 | 0.15 | 0.20 | 0.28 | 0.37 | 0.46 | 0.51 | 0.58 |
| Other CTD-ILD including Sjogren syndrome | 0.13 | 0.21 | 0.29 | 0.36 | 0.51 | 0.65 | 0.74 | 0.82 |
| Systemic Lupus Erythematosus (SLE) or dermatomyositis-ILD | 0.08 | 0.15 | 0.17 | 0.22 | 0.29 | 0.37 | 0.43 | 0.48 |
| Sarcoidosis-ILD | 0.02 | 0.02 | 0.02 | 0.02 | 0.04 | 0.05 | 0.05 | 0.05 |
| ILD due to external factors | 0.03 | 0.07 | 0.09 | 0.10 | 0.16 | 0.17 | 0.21 | 0.23 |
| Unclassifiable ILD | 1.36 | 2.08 | 2.50 | 3.11 | 3.62 | 4.25 | 4.67 | 5.20 |
| Non-progressive fibrosing-interstitial lung disease | | | | | | | | |
| Hypersensitivity pneumonitis-ILD | 0.05 | 0.06 | 0.07 | 0.06 | 0.04 | 0.03 | 0.05 | 0.02 |
| Autoimmune diseases-ILD | 4.59 | 5.36 | 5.38 | 4.94 | 4.24 | 4.68 | 5.02 | 5.23 |
| Rheumatoid arthritis-ILD | 3.75 | 4.56 | 4.56 | 4.15 | 3.57 | 4.07 | 4.49 | 4.63 |
| Systemic sclerosis-ILD | 0.33 | 0.31 | 0.29 | 0.26 | 0.23 | 0.22 | 0.17 | 0.17 |
| Other CTD-ILD including Sjogren syndrome | 0.41 | 0.40 | 0.46 | 0.42 | 0.32 | 0.38 | 0.32 | 0.39 |
| Systemic Lupus Erythematosus (SLE) or dermatomyositis-ILD | 0.24 | 0.24 | 0.24 | 0.25 | 0.21 | 0.18 | 0.15 | 0.13 |
| Sarcoidosis-ILD | 0.02 | 0.02 | 0.02 | 0.04 | 0.02 | 0.01 | 0.03 | 0.03 |
| ILD due to external factors | 0.20 | 0.27 | 0.23 | 0.22 | 0.22 | 0.21 | 0.20 | 0.20 |
| Unclassifiable ILD | 5.53 | 6.06 | 6.10 | 5.96 | 6.15 | 5.68 | 5.77 | 6.30 |

Abbreviations: F-ILD, fibrosing-interstitial lung disease; IPF, idiopathic pulmonary fibrosis; CTD, connective tissue disease

Supplementary Table 3. Progression rate of fibrosing-interstitial lung disease by subtype, 2011-2018

| Subtype | Progression rate (%) | | | | | | | |
| --- | --- | --- | --- | --- | --- | --- | --- | --- |
| Year | 2011 | 2012 | 2013 | 2014 | 2015 | 2016 | 2017 | 2018 |
| Overall | 19.10 | 23.88 | 27.69 | 33.46 | 39.84 | 44.12 | 45.38 | 46.93 |
| Hypersensitivity pneumonitis-ILD | 32.43 | 36.17 | 42.62 | 53.62 | 75.00 | 77.63 | 67.90 | 85.07 |
| Autoimmune diseases-ILD | 17.86 | 21.77 | 25.71 | 32.33 | 42.89 | 45.05 | 45.60 | 48.08 |
| Rheumatoid arthritis-ILD | 16.78 | 18.94 | 22.17 | 28.23 | 38.40 | 39.17 | 38.66 | 41.29 |
| Systemic sclerosis-ILD | 19.51 | 32.31 | 41.37 | 52.17 | 62.01 | 68.12 | 75.14 | 77.08 |
| Other CTD-ILD including Sjogren syndrome | 24.44 | 34.95 | 39.01 | 46.72 | 61.41 | 63.12 | 69.67 | 67.85 |
| Systemic Lupus Erythematosus (SLE) or dermatomyositis-ILD | 24.22 | 38.58 | 40.78 | 46.44 | 57.94 | 67.51 | 74.75 | 78.03 |
| Sarcoidosis-ILD | 54.55 | 42.86 | 50.00 | 37.50 | 66.67 | 80.00 | 58.54 | 68.29 |
| ILD due to external factors | 11.61 | 20.47 | 28.57 | 32.10 | 42.78 | 45.36 | 51.64 | 53.60 |
| Unclassifiable ILD | 20.00 | 25.59 | 29.09 | 34.28 | 37.06 | 42.79 | 44.75 | 45.24 |

Abbreviations: ILD, interstitial lung disease; CTD, connective tissue disease

Supplementary Table 4. Incidence of fibrosing interstitial lung disease by year according to subtype, 2012-2018

| Subtype | Incidence rate per 100,000 | | | | | | |
| --- | --- | --- | --- | --- | --- | --- | --- |
|  | 2012 | 2013 | 2014 | 2015 | 2016 | 2017 | 2018 |
| Progressive fibrosing-interstitial lung disease (PF-ILD) | | | | | | | |
| Hypersensitivity pneumonitis-ILD | 0.022 | 0.030 | 0.032 | 0.043 | 0.024 | 0.018 | 0.020 |
| Autoimmune diseases-ILD | 0.862 | 0.810 | 0.973 | 1.300 | 1.356 | 1.242 | 1.437 |
| Rheumatoid arthritis-ILD | 0.643 | 0.634 | 0.763 | 0.997 | 1.072 | 0.963 | 1.187 |
| Systemic sclerosis-ILD | 0.082 | 0.077 | 0.089 | 0.104 | 0.110 | 0.092 | 0.082 |
| Other CTD-ILD including Sjogren syndrome | 0.116 | 0.095 | 0.129 | 0.183 | 0.179 | 0.180 | 0.152 |
| Systemic Lupus Erythematosus (SLE) or dermatomyositis-ILD | 0.070 | 0.040 | 0.059 | 0.087 | 0.079 | 0.082 | 0.070 |
| Sarcoidosis-ILD | 0.002 | 0.008 | 0.006 | 0.016 | 0.022 | 0.008 | 0.018 |
| ILD due to external factors | 0.048 | 0.058 | 0.053 | 0.075 | 0.073 | 0.084 | 0.064 |
| Unclassifiable ILD | 1.070 | 0.908 | 1.181 | 1.292 | 1.372 | 1.240 | 1.542 |
| Non-progressive fibrosing-interstitial lung disease | | | | | | | |
| Hypersensitivity pneumonitis-ILD | 0.0459 | 0.0556 | 0.0455 | 0.0296 | 0.0275 | 0.0430 | 0.0195 |
| Autoimmune diseases-ILD | 4.1132 | 4.0942 | 3.6991 | 3.0478 | 3.6352 | 4.1187 | 4.2264 |
| Rheumatoid arthritis-ILD | 3.7460 | 3.7207 | 3.3292 | 2.7956 | 3.3388 | 3.7979 | 3.8789 |
| Systemic sclerosis-ILD | 0.1397 | 0.1371 | 0.1147 | 0.0847 | 0.1040 | 0.0900 | 0.0761 |
| Other CTD-ILD including Sjogren syndrome | 0.2096 | 0.2503 | 0.2097 | 0.1221 | 0.0196 | 0.2172 | 0.2538 |
| Systemic Lupus Erythematosus (SLE) or dermatomyositis-ILD | 0.0898 | 0.1013 | 0.1167 | 0.0729 | 0.0923 | 0.0841 | 0.0722 |
| Sarcoidosis-ILD | 0.0200 | 0.0159 | 0.0316 | 0.0099 | 0.0098 | 0.0235 | 0.0195 |
| ILD due to external factors | 0.2175 | 0.1828 | 0.1602 | 0.1714 | 0.1570 | 0.1663 | 0.1542 |
| Unclassifiable ILD | 4.5962 | 4.5948 | 4.2114 | 4.4545 | 4.1396 | 4.1892 | 4.6091 |

Abbreviations: ILD, interstitial lung disease; CTD, connective tissue disease

Supplementary Table 5. Mortality of fibrosing interstitial lung disease by year according to subtype, 2012-2018

|  | Mortality rate (%) | | | | | | | |
| --- | --- | --- | --- | --- | --- | --- | --- | --- |
|  | 2012 | 2013 | 2014 | 2015 | 2016 | 2017 | 2018 | |
| Progressive fibrosing-interstitial lung disease (PF-ILD) | | | | | | | |  |
| Hypersensitivity pneumonitis-ILD | 11.76 | 0.00 | 16.22 | 11.67 | 8.47 | 10.91 | 5.26 | |
| Autoimmune diseases-ILD | 13.85 | 11.80 | 12.44 | 9.86 | 11.17 | 11.73 | 10.51 | |
| Rheumatoid arthritis-ILD | 16.01 | 13.85 | 13.99 | 11.81 | 13.07 | 14.20 | 12.74 | |
| Systemic sclerosis-ILD | 9.46 | 4.85 | 10.42 | 3.66 | 5.96 | 6.46 | 7.09 | |
| Other CTD-ILD including Sjogren syndrome | 7.41 | 8.72 | 9.19 | 6.51 | 8.43 | 7.39 | 5.95 | |
| Systemic Lupus Erythematosus (SLE) or dermatomyositis-ILD | 7.89 | 7.14 | 9.01 | 4.11 | 6.95 | 6.31 | 6.12 | |
| Sarcoidosis-ILD | 0.00 | 9.09 | 0.00 | 5.56 | 8.33 | 8.33 | 10.71 | |
| ILD due to external factors | 11.43 | 17.39 | 15.38 | 25.30 | 21.59 | 19.09 | 18.49 | |
| Unclassifiable ILD | 14.78 | 15.49 | 13.24 | 12.57 | 14.75 | 14.20 | 13.79 | |
| Non-progressive fibrosing-interstitial lung disease | | | | | | | |  |
| Hypersensitivity pneumonitis-ILD | 3.33 | 2.86 | 3.13 | 0.00 | 5.88 | 0.00 | 0.00 | |
| Autoimmune diseases-ILD | 4.00 | 4.67 | 5.42 | 5.19 | 3.80 | 3.26 | 2.65 | |
| Rheumatoid arthritis-ILD | 4.35 | 4.94 | 6.02 | 5.66 | 4.09 | 3.39 | 2.69 | |
| Systemic sclerosis-ILD | 2.58 | 4.11 | 4.55 | 3.42 | 1.82 | 2.30 | 1.14 | |
| Other CTD-ILD including Sjogren syndrome | 2.99 | 3.86 | 0.95 | 0.61 | 1.03 | 3.03 | 3.52 | |
| Systemic Lupus Erythematosus (SLE) or dermatomyositis-ILD | 0.83 | 2.46 | 3.91 | 4.72 | 2.22 | 1.33 | 0.00 | |
| Sarcoidosis-ILD | 0.00 | 9.09 | 0.00 | 0.00 | 0.00 | 0.00 | 7.69 | |
| ILD due to external factors | 6.62 | 10.43 | 12.73 | 13.51 | 4.72 | 3.88 | 2.91 | |
| Unclassifiable ILD | 5.70 | 5.87 | 6.14 | 4.69 | 4.89 | 5.21 | 3.96 | |

Abbreviations: ILD, interstitial lung disease; CTD, connective tissue disease

Supplementary Table 6. Number and proportion of patients by subtype in PF-ILD other than IPF. ICD-10 code in the year of the first diagnosis of PF-ILD was used as a criterion for subtype determination. 2018

| Subtype | Progressive fibrosing-interstitial lung disease (PF-ILD) |
| --- | --- |
|  | n=5,325 (%) |
| Hypersensitivity pneumonitis-ILD | 56 (1.05) |
| Autoimmune diseases-ILD | 2,695 (50.61) |
| Rheumatoid arthritis-ILD | 1,992 (37.41) |
| Systemic sclerosis-ILD | 273 (5.13) |
| Other CTD-ILD including Sjogren syndrome | 397 (7.46) |
| Systemic Lupus Erythematosus (SLE) or dermatomyositis-ILD | 206 (3.87) |
| Sarcoidosis-ILD | 39 (0.73) |
| ILD due to external factors | 131 (2.46) |
| Unclassifiable ILD | 2,448 (45.97) |

| Abbreviations: PF-ILD, progressive fibrosing-interstitial lung disease; IPF, idiopathic pulmonary fibrosis |  |  |  |  |
| --- | --- | --- | --- | --- |
